# CmGID1A-RGL1 GA-Dependent Interaction Orchestrates Flowering in Chrysanthemum

**DOI:** 10.3390/plants15111660

**Published:** 2026-05-28

**Authors:** Wenwen Liu, Huilin Yan, Xin Zhao, Palinuer Aiwaili

**Affiliations:** 1 College of Horticulture, Xinjiang Agricultural University, Urumqi 830052, China; liuwenwen202103@126.com (W.L.);; 2 Beijing Key Laboratory of Development and Quality Control of Ornamental Crops, Department of Ornamental Horticulture, China Agricultural University, Beijing 100193, China

**Keywords:** chrysanthemum, gibberellins, DELLA protein, flowering, GID1A

## Abstract

Gibberellins (GAs) are key endogenous hormones regulating chrysanthemum flowering, and Gibberellin INSENSITIVE DWARF1 (GID1) is the core receptor of the gibberellin (GA) signaling pathway. However, the functional mechanism of CmGID1A remains unelucidated. Here, we constructed *CmGID1A*-RNAi silencing lines and characterized the biological function of CmGID1A by phenotypic identification, protein interaction assays, qRT-PCR and RNA-seq. The results of RT-qPCR showed that *CmGID1A* responds to short days and gibberellins. Inhibition of the expression of *CmGID1A* can significantly promote the transition of chrysanthemum from the vegetative growth stage to the reproductive growth stage and accelerate its flowering process. Bimolecular fluorescence complementation (BiFC) and yeast two-hybrid (Y2H) assays confirmed that *CmGID1A* interacts with the DELLA protein CmRGL1 in a gibberellin-dependent manner. RNA-seq results revealed that silencing of *CmGID1A* leads to a significant up-regulation of downstream *Ethylene Response Factor 6* (*ERF6*) expression. Collectively, CmGID1A acts as a GA receptor to mediate GA signal transduction via interacting with CmRGL1, and regulates the expression of *CmERF6* and other downstream genes, thereby participating in the regulation of floral transition in chrysanthemum. This study clarifies the core role of CmGID1A in the GA signaling pathway and provides novel experimental data for enriching the molecular regulatory mechanism of GA in floral transition in chrysanthemum.

## 1. Introduction

Flowering is a crucial developmental transition in the plant life cycle, which is regulated by both external environmental cues and endogenous hormones [1,2]. A variety of transcription factors, such as CONSTANS (CO), as well as receptors including phytochromes and cryptochromes, regulate flowering through five major flowering pathways: the photoperiod pathway, vernalization pathway, gibberellin (GA) pathway, autonomous pathway, and aging pathway [3,4,5,6]. These pathways function both independently and with crosstalk, ultimately integrating flowering signals into key floral regulators such as FLOWERING LOCUS T (FT) and SUPPRESSOR OF OVEREXPRESSION OF CO1 (SOC1), thereby forming a complex gene regulatory network [7,8,9,10].

Gibberellins (GAs) are among the core hormones governing plant growth and development and serve as key endogenous signals regulating flowering [11,12]. The molecular regulatory mechanism of GA signal transduction is a major research focus in floral transition. As a specific GA receptor, GIBBERELLIN INSENSITIVE DWARF1 (GID1) is the initial key component of the GA signaling pathway. Three homologous *GID1* genes, *GID1A*, *GID1B*, and *GID1C*, have been identified in *Arabidopsis* (*Arabidopsis thaliana*) [13]. DELLA proteins, as core negative regulators of the GA signaling pathway, can regulate the expression of hormone response and growth/development-related genes at the transcriptional level by interacting with various transcription factors [14,15,16]. Five DELLA proteins have been identified in *Arabidopsis*, namely REPRESSOR OF GA1-3 (RGA), GIBBERELLIC ACID INSENSITIVE (GAI), RGA-LIKE 1 (RGL1), RGA-LIKE 2 (RGL2) and RGA-LIKE 3 (RGL3) [17]. The GA-dependent interaction between GID1 and DELLA is a core molecular event of GA signal transduction. Upon binding to GA, GID1 undergoes a conformational change, subsequently interacts with DELLA proteins and mediates their ubiquitination and degradation. This relieves the transcriptional repression of GA signaling by DELLA proteins, ultimately initiating the expression of downstream target genes [18,19,20]. This interaction mechanism has been thoroughly analyzed in model plants; however, in non-model plants, the interaction characteristics and functional differentiation of GID1 homologous genes with DELLA proteins and the regulatory network of downstream key transcription factors remain to be systematically verified. Besides gibberellin, ethylene, another classic plant hormone, is also involved in the regulation of plant flowering. In *Arabidopsis thaliana*, Ethylene Response Factor 1 (ERF1) negatively regulates flowering time by inhibiting *FLOWERING LOCUS T* transcription [21]. However, the synergistic effect of different hormones on flowering regulation remains elusive.

Chrysanthemum (*Chrysanthemum morifolium*) is a typical short-day (SD) perennial herbaceous plant with high ornamental and economic value, and its floral transition is a complex physiological process tightly regulated by the integration of exogenous environmental cues (predominantly photoperiod), endogenous phytohormonal signals, and intrinsic developmental programs. Among the various endogenous regulators, GAs play a pivotal role in chrysanthemum flowering, as numerous studies have demonstrated that chrysanthemum flowering is highly dependent on GA signaling [22,23,24,25,26,27]. We previously reported that SDs can regulate GA biosynthesis and GA perception in chrysanthemum, which activates *GID1B* transcription, thereby promoting flowering [28]. However, the downstream response factors of gibberellin signaling regulating flowering in chrysanthemum, as well as the mechanism of its coordinated regulation with other hormones, remain unclear.

This study focused on the gibberellin receptor gene *CmGID1A* in chrysanthemum. We aimed to investigate whether CmGID1A modulates chrysanthemum flowering through interaction with DELLA proteins, identify key downstream flowering-related genes regulated by CmGID1A, and explore the potential crosstalk between CmGID1A-mediated GA signaling and other signaling. This study is expected to advance our comprehensive understanding of the GA-mediated regulatory network underlying chrysanthemum flowering and facilitate the future application of GA signaling in chrysanthemum genetic improvement. We found that CmGID1A interacts with CmRGL1 in a GA-dependent manner, and may ultimately regulate floral transition in chrysanthemum by modulating the ethylene response factor CmERF6. These results clarify the key function of CmGID1A in GA signaling-regulated flowering and its downstream regulatory network in chrysanthemum, providing experimental evidence for the molecular mechanism of GA signaling and its crosstalk with other hormones, and a theoretical basis for the genetic improvement of plant growth and flowering.

## 2. Results

### 2.1. Expression of CmGID1A Responds to Short-Day Conditions and Gibberellins

**Figure 1:**
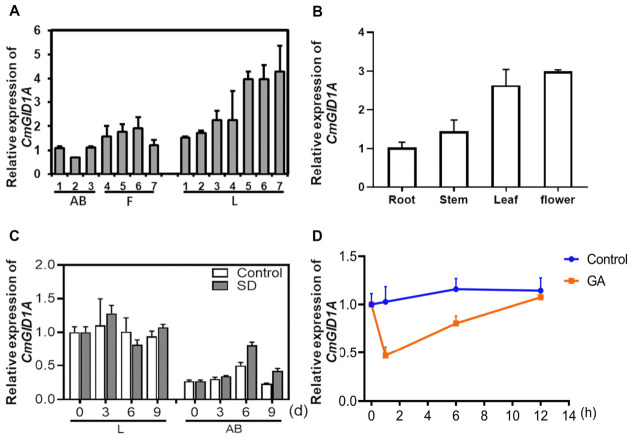
*CmGID1A* responds to short-day photoperiod and GA. (**A**) Relative *CmGID1A* expression analyzed by RT-qPCR in chrysanthemum from vegetative growth to flowering. AB, Apical bud; F, Flower; L, Leaf; 1, Seedling stage; 2, Vegetative growth stage; 3, Flower bud differentiation stage; 4, Early budding stage; 5, Budding stage; 6, Flower bud coloration stage; 7, Full flowering stage. (**B**) Relative *CmGID1A* expression analyzed by RT-qPCR in different organs of chrysanthemum. (**C**) Relative *CmGID1A* expression analyzed by RT-qPCR in leaves and apical buds of chrysanthemum plants grown under long-day (LD) conditions for 60 d and then transferred to short-day (SD) conditions or maintained under LDs as control. (**D**) Relative *CmGID1A* expression analyzed by RT-qPCR in chrysanthemum after a single treatment with 100 µM GA4 + 7 or 3% (*v*/*v*) dimethyl sulfoxide (DMSO) only as control for 14 h. *UBIQUITIN* (*UBI*) was used as an internal control. The results are the means of 3 biological replicates with standard deviation.

To investigate how short-day (SD) conditions induce gibberellins (GAs) to promote flowering in chrysanthemum, we identified the GA-receptor GA INSENSITIVE DWARF1 A (CmGID1A) in chrysanthemum and detected the expression of *CmGID1A* in apical buds and leaves at different developmental stages by RT-qPCR. The results showed that the expression of *CmGID1A* was low in apical buds and leaves at the seedling and vegetative growth stages. After entering the floral bud differentiation stage, its expression gradually increased and peaked from the squaring stage to the flower bud coloration stage, with a marked increase in leaves (Figure 1A). This indicates that the expression of *CmGID1A* is significantly up-regulated during development and plays a key role, especially in the reproductive stage. Analysis of the organ-specific expression of *CmGID1A* revealed high expression in leaves and flowers (Figure 1B), suggesting that *CmGID1A* participates in floral transition in chrysanthemum.

To examine whether the expression of *CmGID1A* is affected by SDs, we measured the expression of *CmGID1A* in leaves and apical buds of chrysanthemum plants grown under long-day (LD) conditions for 60 d and then transferred to SDs or maintained under LDs (as a control). Compared with the control, the expression of *CmGID1A* significantly increased in SD treatment for 6–9 d in apical buds, whereas no obvious change was observed in leaves (Figure 1C), demonstrating that the expression of *CmGID1A* is induced by SDs, and is more sensitive in apical buds, likely mediating SDs-induced floral transition in chrysanthemum.

To explore the effect of gibberellins on *CmGID1A*, we measured the expression of *CmGID1A* in leaves of chrysanthemum plants treated with exogenous GAs. Compared with the control, the expression of *CmGID1A* rapidly decreased within 2 h after GA treatment (to approximately 50% of the control level), then gradually increased and recovered to the control level at 14 h (Figure 1D), indicating that *CmGID1A* is transiently inhibited by GAs.

These data suggest that CmGID1A may participate in SD-induced floral transition in chrysanthemum through the GA pathway.

### 2.2. CmGID1A Regulates Flowering in Chrysanthemum

**Figure 2:**
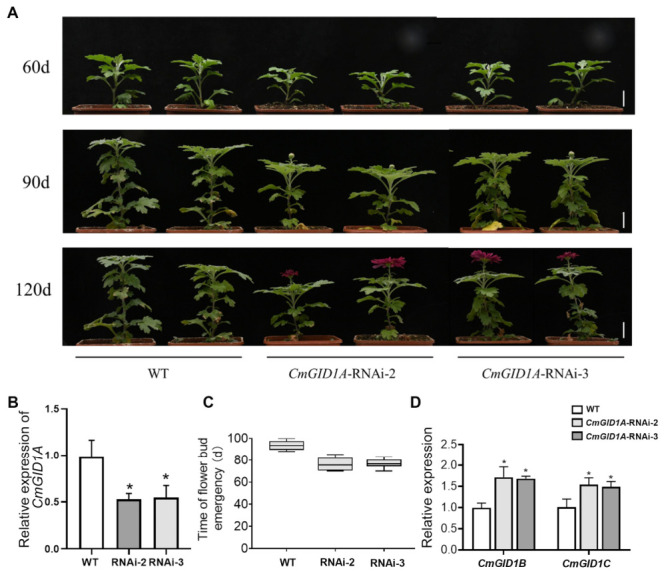
CmGID1A regulates flowering of chrysanthemum. (**A**) Representative phenotypes of *CmGID1A*-RNAi lines. WT and *CmGID1A*-RNAi plants at 60 d, 90 d or 120 d under SDs, including 60 d of LDs and 0 d, 30 d or 60 d of SDs. Scale bars, 2 cm. (**B**) Relative *CmGID1A* expression analyzed by RT-qPCR in WT and *CmGID1A*-RNAi plants grown for 40 d under LDs. (**C**) Time to flower bud emergence of WT and *CmGID1A*-RNAi plants. Six samples were used to calculate the days to flower bud emergence. Center line, median; box limits, upper and lower quartiles; whiskers, 1.5× interquartile range; points, outliers. (**D**) Relative *CmGID1B* and *CmGID1C* expression analyzed by RT-qPCR in WT and *CmGID1A*-RNAi plants grown for 40 d under LDs. *UBI* was used as an internal control for RT-qPCR. The results are the means of 3 biological replicates with standard deviation. Asterisks indicate significant differences according to a Student’s *t*-test in (**B**,**D**) (* *p* < 0.05).

To investigate whether CmG1D1A influences the floral transition, we knocked down *CmGID1A* transcript levels in WT chrysanthemum by generating *CmGID1A*-RNAi (RNA interference) lines (Figure 2B). We grew these lines alongside the wild type (WT) in non-inductive LD conditions for 60 days before transferring them to inductive SD conditions and assessing their flowering phenotype. Flower buds emerged on WT plants after 90 days of growth; in contrast, on the two *CmGID1A*-RNAi transgenic lines after 80 days of growth, 10 days earlier than WT (Figure 2A,C). Fully open flowers were observed on the two *CmGID1A*-RNAi transgenic lines after 120 days, but WT plants remained in bud (Figure 2A), indicating that silencing *CmGID1A* can significantly promote the transition from vegetative growth to reproductive growth, and accelerate the flowering process in chrysanthemum.

Since the chrysanthemum GID1 family comprises three members with functional redundancy, we examined the expression levels of *CmGID1B* and *CmGID1C* in *CmGID1A*-RNAi transgenic lines, and found their expression was significantly elevated in the silenced lines (Figure 2D), suggesting that plants may compensate for the loss of CmGID1A function by upregulating the expression of the other 2 GID1 members to maintain basal homeostasis of GA signaling.

### 2.3. The Interaction Between CmGID1A and CmRGL1 Is Strictly GA-Dependent

**Figure 3:**
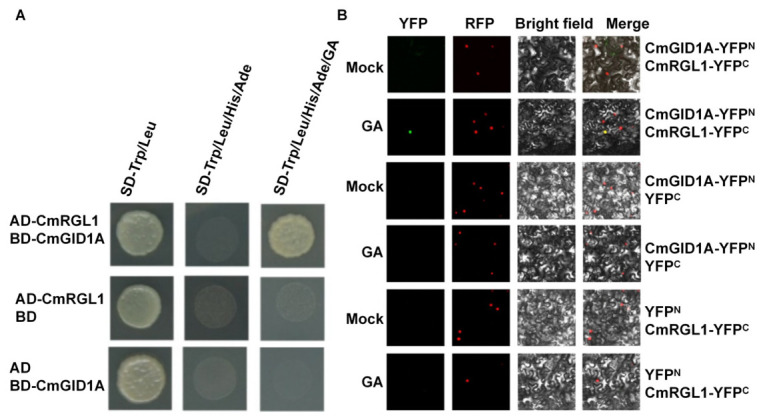
CmGID1A interacts with CmRGL1 in a GA-dependent manner. (**A**) Y2H assays evaluating the interaction between CmGID1A and CmRGL1. The bait BD-CmGID1A and prey AD-CmRGL1 plasmids were cotransferred into yeast strain Y2HGold. Transformants were grown on synthetic defined (SD) medium lacking Leu and Trp (SD-TL) and then transferred to SD medium lacking Leu, Trp, Ade, and His and with 10 µM GA4 + 7. pGADT7 or pGBKT7 as a negative control. (**B**) Interaction of CmGID1A and CmRGL1 in a BiFC assay. *N. benthamiana* leaves were co-infiltrated with CmGID1A-YFP^N^ and CmRGL1-YFP^C^ constructs and visualized by confocal microscopy 3 d after infiltration and sprayed with 100 μM GA4 + 7. Combinations of CmGID1A-YFP^N^ and YFP^C^, and CmRGL1-YFP^C^ and YFP^N^ were used as negative controls. The green fluorescence is the YFP, and the red fluorescence is the nucleus marker RFP (red fluorescent protein with H2B). Scale bars, 100 μm.

Previous studies have shown that DELLA is the key repressor of gibberellin signal transduction, and the GA-GID1-DELLA trimer is the core of gibberellin signaling [18,19,20]. Therefore, we detected the interaction between CmGID1A and DELLAs after gibberellin treatment. The results of the yeast two-hybrid (Y2H) assay showed that the yeast strain co-transformed with BD-CmGID1A and AD-CmRGL1 could only grow normally on the GA-supplemented selective medium (SD-Trp/Leu/His/Ade/GA), but failed to grow on the GA-free selective medium (SD-Trp/Leu/His/Ade) and the empty vector control groups (Figure 3A). In the bimolecular fluorescence complementation (BiFC) assay, we fused CmGID1A to the N-terminal half of yellow fluorescent protein (YFP) and CmRGL1 to the C-terminal half of YFP and co-infiltrated the encoding constructs in *N. benthamiana* leaves. We only observed the reconstitution of YFP fluorescence signals in the nucleus of the co-expression group of CmGID1A-YFP^N^ and CmRGL1-YFP^C^ under GA treatment conditions, as evidenced by colocalization with a nuclear marker, whereas we detected no YFP fluorescence signals in the Mock control and the single vector expression groups (Figure 3B). These results indicate that CmGID1A interacts with CmRGL1 in a GA-dependent manner to initiate GA signal transduction.

### 2.4. Analysis of CmGID1A Downstream Regulation Network

**Figure 4:**
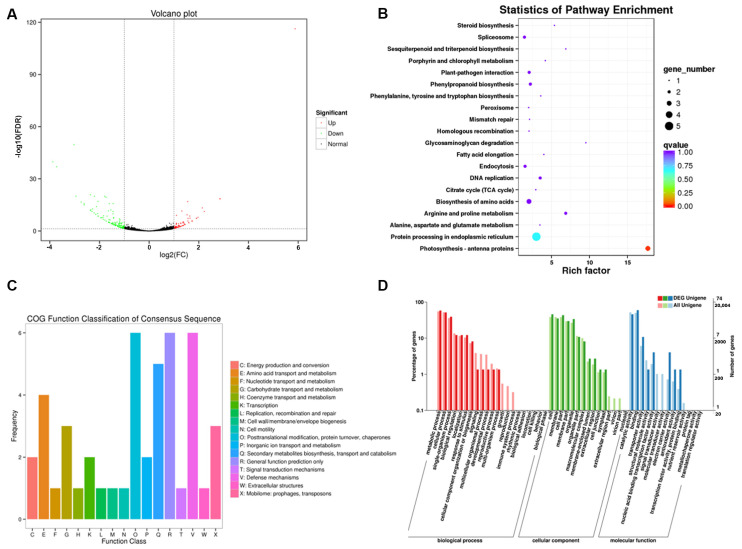
Analysis of CmGID1A downstream regulation network. (**A**) Volcanic map of WT vs. *CmGID1A*-RNAi in shoot apical meristems. The abscissa represents the change in gene expression multiple, and the ordinate represents the significance level of differential genes. Red is upregulated, green is downregulated, and black is non-differentially expressed. Vertical dashed lines indicate genes with a fold change greater than 2, and horizontal dashed lines represent *p*-values < 0.05. (**B**) The scatter plot for pathway enrichment analysis of DEGs. Top statistics of KEGG pathway enrichment for WT vs. *CmGID1A*-RNAi. The Rich Factor is the ratio of differentially expressed gene numbers annotated in this pathway terms to all gene numbers annotated in this pathway term. q ≤ 0.05 is significantly enriched. (**C**) Histogram presentation of the COG classification of the differentially expressed genes in one comparison of WT vs. *CmGID1A*-RNAi in shoot apical meristems. The capital letters in the *x*-axis indicate the COG categories as listed on the right and the *y*-axis indicates the number of DEGs in each category. (**D**) GO functional classification of DEG Unigenes and all Unigenes. Bar chart showing the functional distribution (percentage and number) of differentially expressed genes (DEG Unigenes) and all Unigenes across biological process, cellular component, and molecular function.

In order to show the molecular mechanism of CmGID1A regulating floral transition in chrysanthemum, we identified differentially expressed genes between the leaves of the WT and *CmGID1A*-RNAi plants (obtained from preliminary research) [28] by transcriptome deep sequencing (RNA-seq). We obtained 156 differentially expressed genes (DEGs; using the criteria |Log2[fold-change]| ≥ 1 and q-value 102 ≤ 0.05 for *CmGID1A*-RNAi plants), of which 74 were upregulated and 82 were downregulated relative to the WT (Figure 4A). KEGG (Kyoto Encyclopedia of Genes and Genomes) pathway enrichment analysis of these DEGs indicated that terms associated with ‘photosynthesis—antenna proteins’, ‘protein processing in endoplasmic reticulum’ and ‘biosynthesis of amino acids’ are significantly enriched (Figure 4B). COG (Clusters of Orthologous Groups) function classification of the DEGs indicated terms associated with ‘posttranslational modification, protein turnover, chaperones’, ‘defense mechanisms’ and ‘secondary metabolites biosynthesis, transport and catabolism’ (Figure 4C). GO (Gene Ontology) enrichment classification showed that DEGs were mainly enriched in metabolic processes, cellular processes and stimulus response (biological process), cell, membrane and organelle (cellular component), and catalytic activity and binding (molecular function) (Figure 4D), indicating core roles of CmGID1A in metabolic regulation and environmental adaptation.

### 2.5. CmERF6 Is a Key Downstream Target of CmGID1A-GA Signaling

**Figure 5:**
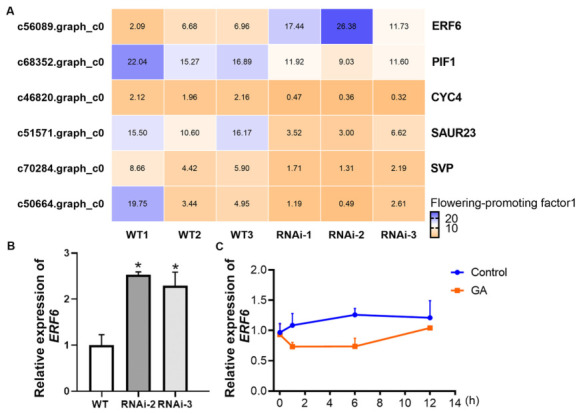
ERF6 is a key downstream target of CmGID1A-GA signaling. (**A**) Heatmap of DEGs includes transcription factors related to hormone and flowering in WT and *CmGID1A*-RNAi plants. Red represents high expression levels. (**B**) Relative *CmERF6* expression analyzed by RT-qPCR in WT and *CmGID1A*-RNAi plants grown for 40 d under LDs. (**C**) Relative *CmERF6* expression analyzed by RT-qPCR in chrysanthemum after a single treatment with 100 µM GA4 + 7 or 3% (*v*/*v*) dimethyl sulfoxide (DMSO) only as control for 14 h. *UBI* was used as an internal control for RT-qPCR. The results are the means of 3 biological replicates with standard deviation. Asterisks indicate significant differences according to a Student’s *t*-test in (**B**) (* p < 0.05).

After further analysis of the transcriptome data, we focused on DEGs annotated as components of the following plant hormone signal or flowering pathways. Heatmap analysis showed that the *CmGID1A*-RNAi lines led to dramatic upregulation of *ERF6* and downregulation of genes, including *PIF1*, *SAUR23*, *SVP*, and flowering-promoting factor 1, compared with WT (Figure 5A). *ERF6* is a key transcription factor of the plant *AP2/ERF* family, which mainly mediates ethylene signal and plays a core regulatory role in disease resistance, stress tolerance, growth and development [29,30,31]. qRT-PCR further verified the significant elevation of *CmERF6* expression in the *CmGID1A*-RNAi lines (Figure 5B), consistent with transcriptomic data. To investigate whether GA regulates *CmERF6*, we detected the expression of *CmERF6* after GA treatment. It was found that within 14 h after GA application, the expression of *CmERF6* was slightly downregulated (Figure 5C), indicating that *CmERF6* was inversely regulated by the CmGID1A/GA signaling pathway and significantly involved in floral transition in chrysanthemum, representing an important downstream factor for dissecting the mechanism by which GA regulates flowering.

## 3. Discussion

Gibberellins (GAs) are pivotal phytohormones that regulate diverse aspects of plant growth and development, including the critical transition from vegetative to reproductive growth [32,33,34]. In this study, we elucidated the functional mechanism of the gibberellin receptor CmGID1A in Chrysanthemum. The results demonstrated that CmGID1A is a key component of gibberellin signaling. As a gibberellin receptor, CmGID1A interacts with the DELLA protein CmRGL1 in a gibberellin-dependent manner, regulating the expression of downstream hormone and flowering-related genes, such as *CmERF6*, and may cooperate with ethylene signaling to regulate the flowering process of chrysanthemum. This work provides new insights into the molecular interaction between gibberellin and ethylene signaling during the flowering transition in chrysanthemum.

It is well established that chrysanthemum is a typical short-day (SD) plant, and its flowering is tightly coupled with photoperiodic cues and endogenous hormone levels [19]. Our data revealed that *CmGID1A* expression is developmentally regulated, showing low levels during the vegetative stage but a significant upsurge during the reproductive phase, particularly in leaves and apical buds (Figure 1A,B). This organ-specific expression pattern suggests that *CmGID1A* plays a distinct role in floral meristem differentiation. We previously reported that SDs can activate *CmGID1B* transcription via the PHOTOLYASE/BLUE LIGHT RECEPTOR2–CRYPTOCHROME-INTERACTING bHLH1 (PHR2–CIB1) complex in chrysanthemum [28]. Consistent with this, our findings specifically demonstrate that the expression of the GA receptor CmGID1A itself is induced by SD conditions, particularly in the apical buds (Figure 1C). This indicates that the apical bud serves as the primary site for GA signal perception during floral induction. Previous studies have demonstrated that DELLAs directly bind to CO, a key regulator of the photoperiod pathway, and inhibit its transcriptional activity, thereby reducing *FT* expression under long-day conditions (LDs) in *Arabidopsis* [15,16,35]. In our study, CmGID1A interacts with the DELLA protein CmRGL1 (Figure 3), which may further affect the regulation of chrysanthemum flowering mediated by the CO-FT module. Furthermore, exogenous GA application leads to the transient suppression of *CmGID1A* (Figure 1D), which aligns with the classic feedback regulatory mechanism in the GA signaling pathway—wherein activation of the pathway typically triggers downregulation of its receptor to maintain homeostasis [36].

Contrary to the well-known role of GAs in promoting flowering in long-day plants like Arabidopsis, our phenotypic analysis of *CmGID1A*-RNAi lines revealed that silencing this GA receptor accelerates flowering in chrysanthemum (Figure 2A,C). Intriguingly, this early-flowering phenotype occurred alongside a significant upregulation of other GA receptor genes, notably *CmGID1B* and *CmGID1C* (Figure 2D). This suggests a complex regulatory interplay within the chrysanthemum GID1 family. Consistent results were obtained in our previous study concerning CmGID1B [28]. Rather than acting as a direct repressor, the downregulation of *CmGID1A* may trigger a compensatory mechanism. This effect elevates endogenous gibberellin levels and enhances GA sensitivity; meanwhile, reduced *CmGID1A* expression induces the upregulation of functionally dominant homologs such as *CmGID1B,* thereby promoting chrysanthemum flowering. Such functional redundancy and hierarchical compensation underscore the sophisticated regulation of GA signaling in modulating meristem identity in chrysanthemum.

The core mechanism of GA signaling relies on the hormone-dependent interaction between GID1 receptors and DELLA proteins, leading to DELLA degradation [37,38,39]. Consistent with this, our yeast two-hybrid (Y2H) and bimolecular fluorescence complementation (BiFC) assays provided direct evidence that CmGID1A interacts strictly with CmRGL1 in the presence of GA (Figure 3). This confirms that CmGID1A is a functional receptor that adheres to the canonical ‘molecular glue’ mechanism. In the absence of GA, CmRGL1 likely represses transcription factors necessary for flowering. Upon GA binding, CmGID1A captures CmRGL1, targeting it for degradation and thereby derepressing downstream genes.

One of the most significant findings of this study is the identification of CmERF6 as a key downstream target. Transcriptome analysis revealed that CmERF6 is dramatically upregulated in the *CmGID1A*-RNAi plants (Figure 5A,B). This is particularly intriguing because *ERF6* is a member of the AP2/ERF family, which is a central mediator of ethylene signaling and stress responses [40,41]. In Arabidopsis, AtERF1 and AtERF6 have been shown to act as repressors of flowering by directly inhibiting *FT* expression [30]. Our data suggest that *CmGID1A* may play a similar role in chrysanthemum: when *CmGID1A* is silenced, the inhibition of *CmERF6* is relieved, thereby promoting flowering. The reverse regulation of *CmERF6* by gibberellin (Figure 5C) further supports a model in which the gibberellin-CmGID1A signaling pathway typically suppresses the expression of *CmERF6*. This indicates that the early flowering phenotype observed in *CmGID1A*-RNAi might be mediated by the release of the inhibition on *CmERF6*. This interaction suggests that chrysanthemum integrates hormone signals (auxin and ethylene) to precisely regulate its flowering time in response to environmental stress and photoperiodic changes.

**Figure 6:**
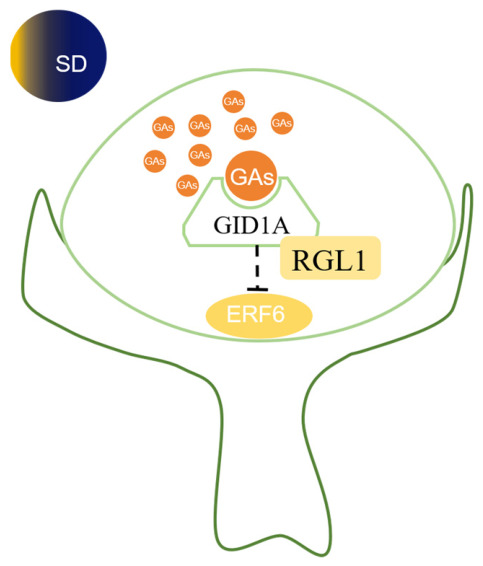
Regulatory model of CmGID1A-CmRGL1 in chrysanthemum flowering. Under short-day conditions, CmGID1 perceives active GAs, interacts with CmRGL1, and indirectly inhibits the expression of *CmERF6*, thereby promoting floral transition in chrysanthemum. Dashed lines represent indirect regulation. SD, short-day condition.

In conclusion, we propose a mechanistic model in which CmGID1A acts as a gibberellin-dependent receptor that physically engages CmRGL1 to orchestrate the transcriptional regulation of downstream target genes—most notably the ethylene-responsive transcription factor CmERF6 (Figure 6).

## 4. Materials and Methods

### 4.1. Plant Materials and Growth Conditions

This study utilized *CmGID1A*-RNAi and WT chrysanthemum (*Chrysanthemum morifolium* ‘Fall Color’). To construct the RNAi vector, a 459-bp sense fragment and its corresponding antisense fragment of *CmGID1A* were cloned into the *pFGC1008* vector via AscI/SwaI and BamHI/PacI restriction sites, respectively. This resulted in an RNA expression vector driven by the *35S* promoter, containing an intron-spliced “hairpin” structure. The recombinant vector was subsequently transformed into *Agrobacterium tumefaciens* strain EHA105. Chrysanthemum leaf discs were used as explants for infection and transformation. Hygromycin-resistant plants were obtained through selection. Primers used are shown in Supplementary Data Set S1.

Forty-day-old plants were transplanted into pots (9 cm in diameter) containing a peat:vermiculite (1:1, *v*/*v*) mixture and grown in a growth chamber. The growth conditions were as follows: temperature 23 ± 1 °C, relative humidity 40%, light intensity 100 μmol·m^−2^·s^−1^, under either a long-day (LD) photoperiod (16 h light/8 h dark) or a short-day (SD) photoperiod (8 h light/16 h dark).

### 4.2. RNA Extraction and RT-qPCR

The fully expanded fourth leaf was harvested from five biological replicates at Zeitgeber time 8 (ZT8). Total RNA was extracted from these samples using RNAiso Plus (TaKaRa, Kusatsu-shi, Japan) following the manufacturer’s instructions. First-strand cDNA was synthesized from 1 μg of total RNA using HiScript II Q RT SuperMix for qPCR (with gDNA wiper) (Vazyme, Nanjing, China). Quantitative real-time PCR (RT-qPCR) was performed on the StepOne Real-Time PCR System (Applied Biosystems, Waltham, MA, USA) under standard cycling conditions, using 2× Realtime PCR Super Mix (SYBR Green, with anti-Taq antibody) (Mei5 Biotechnology Co., Ltd., Beijing, China). Expression of the *CmUBI* gene (GenBank accession: NM_112764) served as an internal reference. Relative transcript levels were calculated using the 2^−ΔΔCT^ method [42]. Primers used are shown in Supplementary Data Set S1.

### 4.3. GA Treatment

WT tissue-cultured seedlings were grown under long-day (LD) conditions for 40 days, then transferred to an LD growth chamber for an additional two weeks. Subsequently, the plants were shifted to short-day (SD) conditions and sprayed with 100 μM GAs. GA4 + 7 was dissolved in 3% (*v*/*v*) DMSO, with an equivalent concentration of DMSO solution serving as the control. During the treatment period, plants were sprayed once every five days for one month.

### 4.4. Yeast Two-Hybrid Assays

The yeast two-hybrid (Y2H) assay was performed using the Matchmaker GAL4 Two-Hybrid System (Clontech, Shiga, Japan). The open reading frame (ORF) sequence of *CmRGL1* was amplified and inserted into the *pGADT7* vector [43] via EcoRI/BamHI restriction sites. The ORF sequence of *CmGID1A* was amplified and inserted into the *pGBKT7* vector [44] via EcoRI/SalI restriction sites. The recombinant plasmids *pGADT7* and *pGBKT7* were co-transformed into the yeast strain Y2HGold. The *GUS* ORF sequence was inserted into either the *pGADT7* or *pGBKT7* vector as a negative control. Transformants were cultured on SD/-Trp-Leu medium, subsequently transferred to SD/-Trp-Leu-His-Ade medium, and then spotted onto plates containing GA for further analysis. Primers used are shown in Supplementary Data Set S1.

### 4.5. BIFC

The open reading frame (ORF) of *CmRGL1* without a terminator was inserted into the *35S-SPYCE(M)* vector via XbaI/KpnI restriction sites, and the ORF of *CmGID1A* was inserted into the *35S-SPYNE(R) 173* vector via XbaI/KpnI restriction sites. Subsequently, the recombinant vectors or control vectors were transformed into *Agrobacterium tumefaciens* strain GV3101. The bacterial suspensions were adjusted to an OD600 of 1.0 using infiltration buffer. Agrobacterium cultures expressing CmGID1A-YFP^N^, CmRGL1-YFP^C^, or control vectors were mixed at a 1:1 volume ratio and infiltrated into leaves of *Nicotiana benthamiana* using a needleless syringe. After 3 days of incubation, GA was sprayed onto the corresponding tobacco leaves prior to imaging. Yellow fluorescent protein (YFP) fluorescence imaging was performed using a Nikon A1 confocal laser scanning microscope (Nikon, Tokyo, Japan). YFP was excited with a 488 nm laser and detected at 525 nm; red fluorescent protein (RFP) was excited with a 561 nm laser and detected at 610 nm. Primers used are shown in Supplementary Data Set S1.

### 4.6. RNA-Seq Analysis

WT and *CmGID1A*-RNAi tissue-cultured seedlings were grown under long-day (LD; 16 h light/8 h dark) conditions for 40 days, then transferred to an LD growth chamber (same photoperiod) for an additional 2 weeks, followed by a 2-week exposure to short-day (SD; 8 h light/16 h dark) conditions. When the shoot apical meristem had nearly completed differentiation into floral primordia, the fourth fully expanded leaf—counted from the apical meristem—was harvested from three biological replicates for total RNA extraction. RNA sequencing was performed on the Illumina HiSeq 2000 platform at Novogene Co., Ltd. (Beijing, China; http://www.novogene.com/). RNA-seq data were processed, assembled, and annotated as previously described [45].

### 4.7. Statistical Analysis

All experiments included at least three biological replicates. Statistical significance of differences was assessed using one-way analysis of variance (ANOVA) followed by Duncan’s multiple range test for comparisons among ≥3 groups, or two-tailed Student’s *t*-test for pairwise comparisons, as implemented in GraphPad Prism v8.0 (GraphPad Software, San Diego, CA, USA).

## 5. Conclusions

This study clarifies the core function and regulatory mechanism of the GA receptor CmGID1A in the GA signaling pathway and floral transition in chrysanthemum. CmGID1A is a key component of GA signaling, which interacts with the DELLA protein CmRGL1 in a strictly GA-dependent manner to mediate GA signal transition.

*CmGID1A* is highly expressed during the reproductive growth stage of chrysanthemum, responds to short-day conditions and GA, and participates in SD-induced floral transition. Silencing *CmGID1A* significantly promotes floral transition and accelerates flowering in chrysanthemum, accompanied by the up-regulation of *CmGID1B* and *CmGID1C* to compensate for its functional loss. Additionally, silencing *CmGID1A* alters the expression of downstream hormone- and flowering-related genes, particularly the significant up-regulation of *Ethylene Response Factor 6* (*CmERF6*).

In summary, CmGID1A acts as a GA receptor, interacts with the DELLA protein CmRGL1 in a GA-dependent manner, regulates the expression of downstream hormone- and flowering-related genes such as *CmERF6*, and may coordinate with ethylene signaling to jointly regulate the flowering process in chrysanthemum. This study enriches chrysanthemum GA signal regulatory networks, provides gene targets and a theoretical basis for genetic engineering of chrysanthemum flowering and agronomic traits, and offers references for GID1 family functional differentiation and GA signaling in non-model plants.

## Data Availability

The original contributions presented in this study are included in the article/Supplementary Material. Further inquiries can be directed to the corresponding author.

## Supplementary Materials

The following supporting information can be downloaded at: https://www.mdpi.com/article/10.3390/plants15111660/s1, Supplementary Data Set S1. List of primers used in this study.
