# Supplementary material for: CmGID1A-RGL1 GA-Dependent Interaction Orchestrates Flowering in Chrysanthemum
Source: Plants (Basel). 2026 May 28;15(11):1660. doi: 10.3390/plants15111660 (PMC13259077; doi:10.3390/plants15111660)
Supplement: Supplementary file 1 [file plants-15-01660-s001.zip › plants-4311825-supplementary.pdf]

Supplementary Data Set 1. List of primers used in this study.

Primer Name	Primer Sequence(5'→3')	Construct
Chrysanthemum Transformation		
GID1A-AscI-F	ttacaattaccatggggcgcgccTAGAGGGTACATCCCCAACC	RNAi-CmGID1A
GID1A-SwaI-R	catgttcatctggggatttaaatATGTTATTTGGTTCTGTTGCT	
GID1A-BamHI-F	cgatctctttgatggggatccATGTTATTTGGTTCTGTTGCT	
GID1A-PacI-R	gactctagggactagttaattaaTAGAGGGTACATCCCCAACC	
Yeast two-hybrid assays		
RGL1-EcoRI-F	gccatggaggccagtgaattcATGAGTCCTCACACTCACAC	pGADT7-CmRGL1
RGL1-BamHI-R	cagctcgagctcgatggatccTTATTGAGAATGGTTTATGTCA	
GID1A-EcoRI-F	atggccatggaggccgaattcATGATGAGAACAATTGGATA	pGBKT7-CmGID1A
GID1A-SalI-R	atgcggccgctgcaggtcgacCTATTCACATTCGGATGTTGG	
BIFC		
RGL1-XbaI-F	gagaacacgggggactctagaATGAGTCCTCACACTCACAC	35S-SPYCE(M)-CmRGL1
RGL1-KpnI-R	gtacatcccgggagcggtaccTTGAGAATGGTTTATGTCA	
GID1A-XbaI-F	gagaacacgggggactctagaATGATGAGAACAATTGGATA	35S-SPYNE(R)173-CmGID1A
GID1A-KpnI-R	ctccatcccgggagcggtaccTTCACATTCGGATGTTGG	
RT-qPCR		
UBI-F	AGCTGAGCAGACTCCCGATG	
UBI-R	AGGCGAATCATCAGTACCAAGT	
GID1A-F	TAATTTCATGTGCCACCGCC	
GID1A-R	TCCTGGCGTCCTCTCATTTG	
GID1B-F	TGGGATATCTCTCGGGCAGG	
GID1B-R	GAGACACCCTCTTCAAACATCCA	
GID1C-F	TCTACTTGTGGGATTATGAGAGGA	
GID1C-R	CCATAAACAATCATCAAAAACCCCT	
ERF6-F	GAAGCTGGAAACTTTGAAGCT	
ERF6-R	AAATGCTTCTTAAGGTTAGT	
